# Two pilot case studies for indirect bridge scour monitoring using low-cost remote sensing for river flow characterisations

**DOI:** 10.12688/openreseurope.19083.2

**Published:** 2025-11-28

**Authors:** Eleonora Perugini, Enrico Tubaldi

**Affiliations:** 1 Civil and Environmental Engineering, University of Strathclyde Faculty of Engineering, Glasgow, Scotland, G1 1XJ, UK

**Keywords:** Bridge-scour; Filed Monitoring; Remote Sensing; Low-cost sensors

## Abstract

Bridge scour is a leading cause of bridge failures worldwide, exacerbated by climate change and increasing flood risks. Real-time data collection plays a critical role in effective flood risk management and decision-making, ultimately enhancing infrastructure resilience. The EU-funded RAMOBRIS (Risk Assessment and Monitoring for Bridges under Scour Hazard) project investigated cost-effective monitoring approaches to develop a novel, multidisciplinary strategy for assessing the risk of critical bridges exposed to scour. This manuscript outlines the monitoring strategy developed during the project, with a focus on the application of cost-effective sensors for hydraulic monitoring. The adopted methodology employs an indirect approach using low-cost remote sensing sensors to assess hydraulic properties and estimate scour depth through advanced formulas. Two pilot case studies were conducted on high-risk masonry bridges over the River Nith in Scotland. Various sensors were installed to evaluate their effectiveness in capturing hydraulic data and monitoring scour dynamics. Data from low-cost sensors were evaluated against data collected from higher-cost sensors or other available datasets. The results showed that low-cost sensors for measuring water levels provided accuracy comparable to high-cost radar systems, while being more cost-effective and easier to install. Video data from solar cameras enabled extensive measurements of surface velocity and discharge, improving the understanding of flow dynamics. The study confirmed the feasibility of using image velocimetry techniques for long-term estimation of river velocity and discharge, although further validation is required. These findings highlight the potential of low-cost and innovative sensor technologies. The open-access dataset generated in this study, which will be periodically updated with new data, provides a valuable resource of real-world information for ongoing research in hydraulic monitoring and bridge safety assessment.

## Plain language summary

Bridge scour, the erosion of riverbed material around bridge foundations, is currently a major cause of bridge failures worldwide. This problem is expected to worsen due to the increased intensity and frequency of precipitation driven by climate change, leading to higher flood risks. To improve flood risk management and enable data-informed decision-making to protect infrastructure, it is crucial to develop cost-efficient monitoring strategies. This study outlines the monitoring strategy tested during the EU-funded RAMOBRIS (Risk Assessment and Monitoring for Bridges under Scour Hazard) project, which relies on the use of low-cost technologies to measure hydraulic properties and then uses them to estimate scour depth through advanced formulas. The manuscript also describes the two pilot studies conducted to evaluate the proposed approach, where various sensors were installed, with a specific focus on analysing the data collected from the low-cost sensors used for hydraulic monitoring. The comparison between the water levels obtained from low-cost sensors and those obtained from more expensive radar systems showed that the former can provide accuracy comparable to that of the more expensive systems, but with the advantage of being more affordable and easier to install. The study also explored the use of image velocimetry techniques, which involve measuring the surface velocity of river water by analysing videos, confirming that these methods could be used for long-term assessments of river velocity and flow, encouraging further research in this field. In summary, this research highlights the feasibility of using low-cost sensors for hydraulic monitoring and bridge safety, promoting their use for indirect scour monitoring. The study also establishes an open-access dataset, which will be regularly updated, providing valuable real-world data for investigating flood risk.

## 1 Introduction

Bridges are crucial components of transportation networks, facilitating the efficient movement of goods and people. Their functionality and stability are influenced by various natural hazards, such as earthquakes, landslides, wildfires, and floods, and human-induced hazards, including collisions, overloading, and explosions ( Mei & Guo, 2024). Among these, hydraulic hazards represent the leading cause of bridge failures globally. This is underscored by Zhang *et al.* (2022), who conducted a statistical analysis of ten prior studies encompassing over 4,500 bridge failure events. Bridges crossing rivers are subjected to various actions, such as hydraulic load, flood-induced scour and debris accumulation ( Burghardt *et al.*, 2025; Carnacina *et al.*, 2019; Kabir *et al.*, 2023). Particularly, scour, the erosion of river sediments due to the action of the river water, can lead to the formation of deep holes around the bridge foundation, which can compromise the load-bearing capacity of a bridge, potentially resulting in structural failure or collapse.

Scour is generally classified into three primary categories: general scour, contraction scour, and local scour. General scour affects the entire area surrounding a bridge and is primarily a consequence of natural riverbed erosion during high flow conditions, independent of any structures present. In contrast, contraction scour occurs when the river channel narrows due to the presence of a structure, leading to an increase in water velocity and subsequent sediment removal. This type of scour is associated with the presence of bridge piers or abutments, which reduce the cross-sectional area and significantly influence flow dynamics, intensifying sediment erosion. Local scour, the third category, is induced by the interaction of water flow with the bridge structure, resulting in turbulence and vorticity that increase shear stress on the sediment. This type of scour is often the most critical, as it can lead to the creation of a scour hole around the bridge foundation, which may deepen over time and pose a serious risk to the structure’s stability.

Bridge scour is currently one of the leading causes of bridge failure worldwide, resulting in significant economic losses and loss of life ( Pizarro *et al.*, 2020), and its impact is expected to increase due to climate change ( Sasidharan *et al*., 2023). Dottori *et al.* (2023) estimate that river flooding currently causes approximately €7.6 billion in annual damages and exposes around 160,000 people per year to inundation across the European Union and the UK. In a 3°C global warming scenario, and without climate adaptation measures, annual flood damage in Europe is projected to rise to €44 billion, with nearly half a million Europeans exposed to flooding each year by the end of the century.

Despite advancements in engineering methodologies and technologies for assessing the safety of waterway bridges, many of these methods depend on expensive sensors or involve costly and complex installations ( Link *et al.*, 2020; Lu *et al.*, 2008; Wang *et al.*, 2017). As a result, current flood risk assessment and emergency management practices often rely on visual inspections and simplistic empirical formulas to estimate scour. However, these approaches are frequently inadequate, especially under rapidly changing conditions during flood events, highlighting the need for more robust and effective monitoring strategies ( Tubaldi *et al.*, 2022). Scour holes can develop gradually over time and then rapidly deepen during severe weather events, potentially leading to sudden structural failure or collapse. Consequently, the risk of bridge scour failure is highest during intense flood events, which are also the most challenging to monitor. Moreover, inspections by divers cannot be conducted until the flood has receded, making them inadequate for assessing bridge safety.

To address these challenges, the EU-funded RAMOBRIS (Risk Assessment and Monitoring for Bridges under Scour Hazard) project investigated novel, low-cost approaches and strategies for monitoring and evaluating the scour risk of critical bridges ( www.ramobris.eu). The outcomes of the study support the implementation of scour monitoring systems, contributing to improved flood risk management, more informed decision-making, and enhanced early warning capabilities, while strengthening the resilience and security of critical infrastructure. As part of the project, two case studies were developed at critical bridges in Scotland, where various sensors were installed to assess the effectiveness of low-cost monitoring strategies.

This paper describes the two pilot case studies, including the installation of instruments and the adopted strategy, with a focus on hydraulic monitoring. The deployed sensors range from expensive to low-cost options and include both underwater installations and remote sensing technologies. The establishment of the two monitoring sites enables the comparison of different monitoring strategies and techniques, while also providing insights into the practical aspects of on-site installation. Furthermore, the data collected from these sites constitute a rich dataset that forms the foundation for further analysis and contributes to the understanding of scour dynamics and bridge performance under varying flow conditions. The data obtained from these case studies are also being used in other research within the same project, aimed at developing new tools for scour risk assessment and decision support systems (DSS) based on observed data ( Perugini *et al.*, 2024). Ultimately, this research seeks to enhance the resilience of bridge infrastructure in the face of climate change and increasing flood risks by suggesting tools and strategies to improve flood early warning systems as well.

The document is organised as follows: Section 2 presents the principles guiding the design of the two pilot case studies and the definition of the adopted monitoring approach. Section 3 outlines the methodology employed, including site selection and the specific sensors deployed. Section 4 provides a detailed description of the two pilot case studies, while Section 5 analyses the hydraulic data obtained from the sensors. Finally, the paper concludes with a summary of the findings and recommendations for future research and practice in the field of bridge scour monitoring and management.

## 2 Bridge-scour monitoring strategies

Establishing effective monitoring strategies for bridge scour is essential for several reasons. First, field monitoring provides real-world data crucial for understanding the complex processes of scour, the mechanisms leading to bridge instability, and the impacts of climate change. Additionally, it enables the detection of maximum scour conditions, which are critical for assessing infrastructure safety and providing early warnings. Secondly, the availability of high-quality monitoring data is fundamental for calibrating and validating numerical models, as well as for training and testing AI-based methods ( Azma *et al*., 2024; Kumar *et al.,* 2023; Tubaldi *et al.*, 2023). Furthermore, monitoring data are necessary for designing new structures, planning effective mitigation measures, and ensuring that risk assessment and asset management strategies are based on reliable data. Despite its importance, many streams and rivers worldwide remain inadequately gauged, partly due to the costs associated with the installation and maintenance of monitoring systems. As a result, the hydraulic properties of many rivers are often unknown, and the characteristics of extreme flood events remain poorly understood. For instance, during the 2022 flood in the Marche region of Italy, monitoring sensors were washed away, leaving no recorded measurements of peak flow rates or water levels. This highlights the urgent need for resilient and comprehensive monitoring solutions capable of capturing critical data even during extreme events.

An effective monitoring strategy should incorporate several key characteristics to ensure detailed and reliable data collection while overcoming practical challenges. First, the monitoring system must be adequately accurate to provide reliable measurements and capture the key physical characteristics of the phenomenon being observed. The required accuracy depends on the specific application, but it is crucial to provide uncertainty estimates alongside the measurements. This allows for a comprehensive understanding of the data and enhances its interpretation. Another important aspect is the use of non-contact sensors, which simplify installation and eliminate the need for time-consuming permissions typically required for sensors installed in riverbeds. These systems also reduce maintenance complexity and are less likely to be damaged during floods. Additionally, remote sensing strategies are of paramount importance for supporting data acquisition, especially in areas that are difficult to access. They offer the advantage of gathering data without the need for physical presence at the monitoring location, making the process more efficient. Furthermore, cost-effective solutions are essential to enable the widespread deployment of sensors, thereby improving the coverage and availability of data across larger regions. These solutions also support long-term monitoring applications. Continuous monitoring is crucial as it allows for a more comprehensive description of events, from low-flow conditions to extreme flooding, and facilitates historical analysis and climate change monitoring. This approach also helps overcome the limitations of sporadic measurements, which are often taken only under low-flow conditions or after an event when access to the river is safer. Finally, the ability to transmit data in real-time is vital for facilitating quick decision-making during extreme events. This enables timely actions, such as closing bridges or evacuating areas, in response to critical thresholds, ensuring the safety of infrastructure and communities.

Various approaches can be employed for monitoring bridge scour. A direct method involves measuring changes in the scour hole or bed level using sensors such as scour probes ( Maroni *et al.,* 2020) or sonars ( Rogers *et al*., 2020), although these sensors tend to be expensive and require underwater installation. A second approach is the indirect evaluation of scour by monitoring structural changes, such as using inclinometers or accelerometers to track bridge stability, or employing radars to detect changes in bridge frequency ( Kazemian *et al.*, 2023; Tola *et al.,* 2023; Tubaldi *et al.*, 2023). While these variations may indicate the evolution of scour, they often detect issues too late, after the bridge has already been affected. Another method is to directly measure flow properties, such as water level and velocity, and use these data as input to estimate scour depth through advanced scour formulas or artificial intelligence techniques, thereby indirectly determining the scour depth. Perugini *et al.* (2024) presented a proof of concept for near real-time estimation of scour at a bridge pier by combining low-cost sensors, large-scale image velocimetry techniques, and advanced scour models. Specifically, the flow properties collected by the sensors were used as input for a temporal scour model based on the dimensionless effective flow work ( Link *et al.*, 2020; Pizarro *et al.*, 2017; Pizarro *et al.*, 2022). Kumar *et al.*. (2023) evaluated the performance of two ensemble methods and one standalone machine learning method for predicting time-dependent scour using laboratory data. Similarly, Azma *et al.* (2024) employed experimental data and deep learning algorithms to predict the evolution of scour depth downstream of a submerged weir.

In this work, the latter approach was selected, as recent developments have led to the creation of various low-cost sensors and techniques for hydraulic monitoring. Therefore, in designing the case studies for this work, this indirect approach was employed, aiming to evaluate the feasibility of using these methods to collect data that can subsequently be used to determine scour through advanced temporal formulas.

## 3 Method

The methodological approach adopted in this study began with the identification of suitable bridge sites and the selection of appropriate monitoring technologies. After a thorough search for potential locations and a review of available sensors, two bridges were selected as pilot sites based on their structural characteristics and susceptibility to scour ( Section 3.1). The most suitable instruments were then chosen according to the specific monitoring objectives and site constraints. Following this preliminary assessment, the sensors described in Section 3.2 were installed to establish two pilot case studies ( Section 4), enabling continuous monitoring of the hydraulic and morphological conditions at the selected bridges. The implemented systems were designed to measure parameters such as water level, flow velocity, and bed elevation, providing real-time information on scour processes under varying hydraulic conditions, and to test both low-cost and more expensive sensor technologies. Subsequently, the data collected from the monitoring systems were processed and compared in order to evaluate the performance of the installed technologies.

### 3.1 Case studies

**Figure 1.**
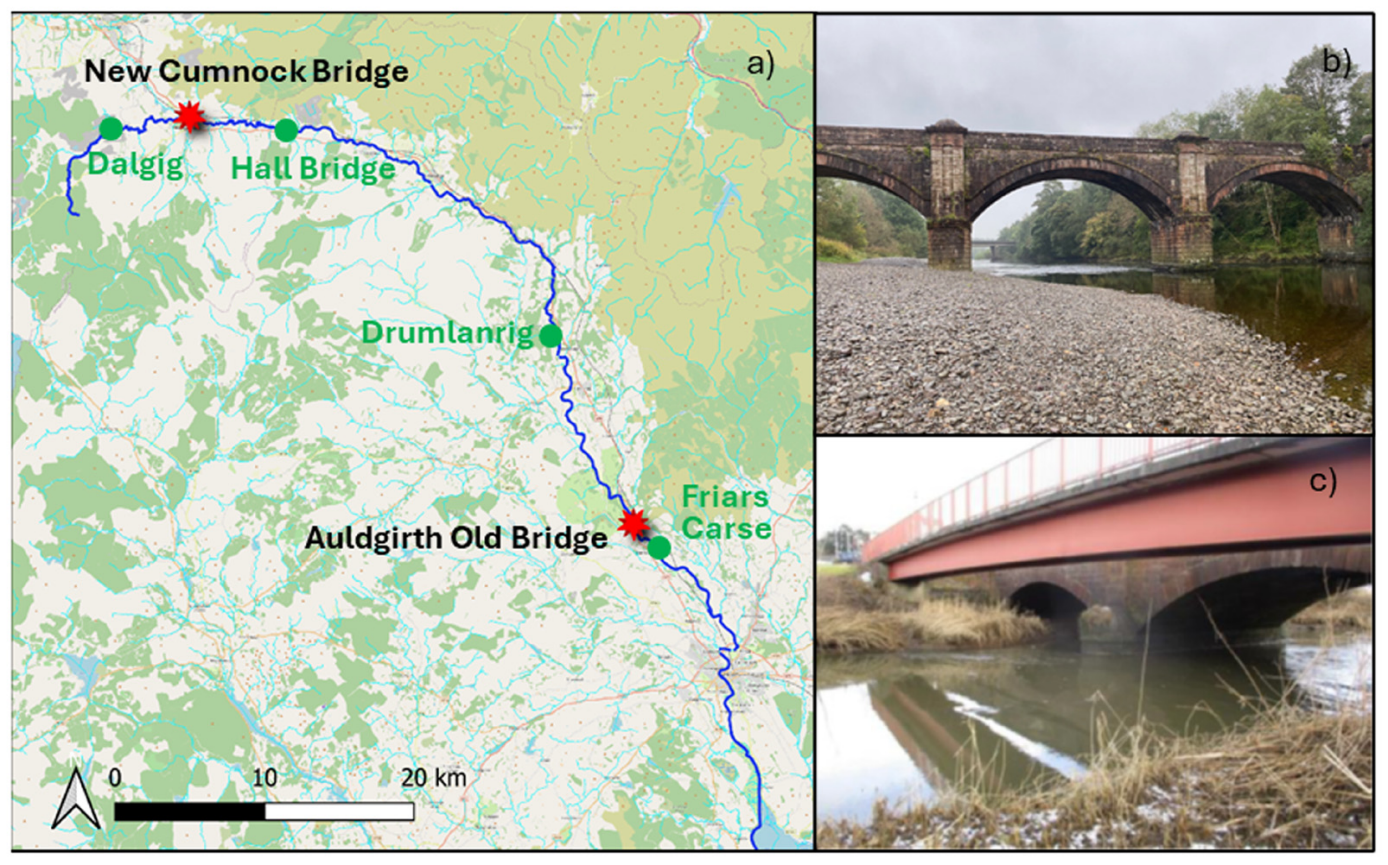
Overview of the case studies. **a**) Case study locations (red dots) and SEPA river gauges locations (green dots). **b**) Auldgirth Old case study. **c**) New Cumnock case study (adapted from Maroni *et al.*, 2020).

The selected case studies were the A76 31 old bridge at Auldgirth (55.1597°N, 3.7100°W) and the A76 200 bridge at New Cumnock (55.4013°N, 4.1829°W). These bridges were chosen based on their classification as high-risk structures for scour by Transport Scotland. Moreover, they are masonry bridges, which are more susceptible to scour due to their shallow foundations. Both sites are located along the River Nith ( Figure 1.a), which originates in East Ayrshire, in the southwest of Scotland, and flows for approximately 110 km before entering the Solway Firth. The river's width ranges from 10 to 30 meters, and its bed consists of fine sand, gravel, and coarse sand sediments. Several SEPA river gauges are situated along the river, measuring water levels and, in some cases, providing discharge estimates using calibrated rating curves ( https://waterlevels.sepa.org.uk/).

The first case study ( Figure 1.b) was selected due to the visible presence of scour hole at the left pier, indicative of ongoing active scour. The structure is a three-span masonry arch bridge, with a span length of 20.10 meters and a width of 4.80 meters. The bridge is now pedestrian-only, as the Auldgirth New Bridge, located just a few dozen meters downstream, handles road traffic. The left pier is situated at the river's edge and is subjected to flow during medium to high water levels, with bed materials consisting of sand and gravel deposits around it. The right pier is located at the centre of the river, where it is supported by a rocky foundation. The SEPA gauge upstream of the bridge is positioned at Drumlanrig, while the downstream gauge is located at Friars Carse.

The second case study ( Figure 1.c) was chosen due to the presence of two scour probes, one on the left pier to monitor local scour and another in the centre of the river to assess general scour ( Maroni *et al.*, 2020). Additionally, the site is easily accessible, with low water levels facilitating river entry. The bridge is a three-span stone masonry arch supporting a two-lane, single-carriageway. Its abutments and piers are founded on spread footings on the natural riverbed. The left pier is situated at the centre of the river, while the right pier is positioned at the river's edge. Immediately upstream of the masonry bridge, there is a pedestrian bridge. This bridge is a single-span, simply supported composite structure, with a clear span of 34 m. In this location, a scaffolding is already in place, which was used for the installation of the scour probes. SEPA river gauges consist of one station upstream of the bridge at Dalgig and another downstream at Hall Bridge.

### 3.2 Equipment

The sensors chosen for installation at the two case studies include: i) Scour probes to monitor changes in the riverbed and track the evolution of the scour hole, which have already been deployed at one of the sites; ii) RiverTrack sensors and Solar Cameras as low-cost sensors for monitoring the hydraulic properties of the river, and iii) a traditional radar system, used to validate the data collected from the low-cost sensors. A detailed description of these sensors is provided below.

**Figure 2.**
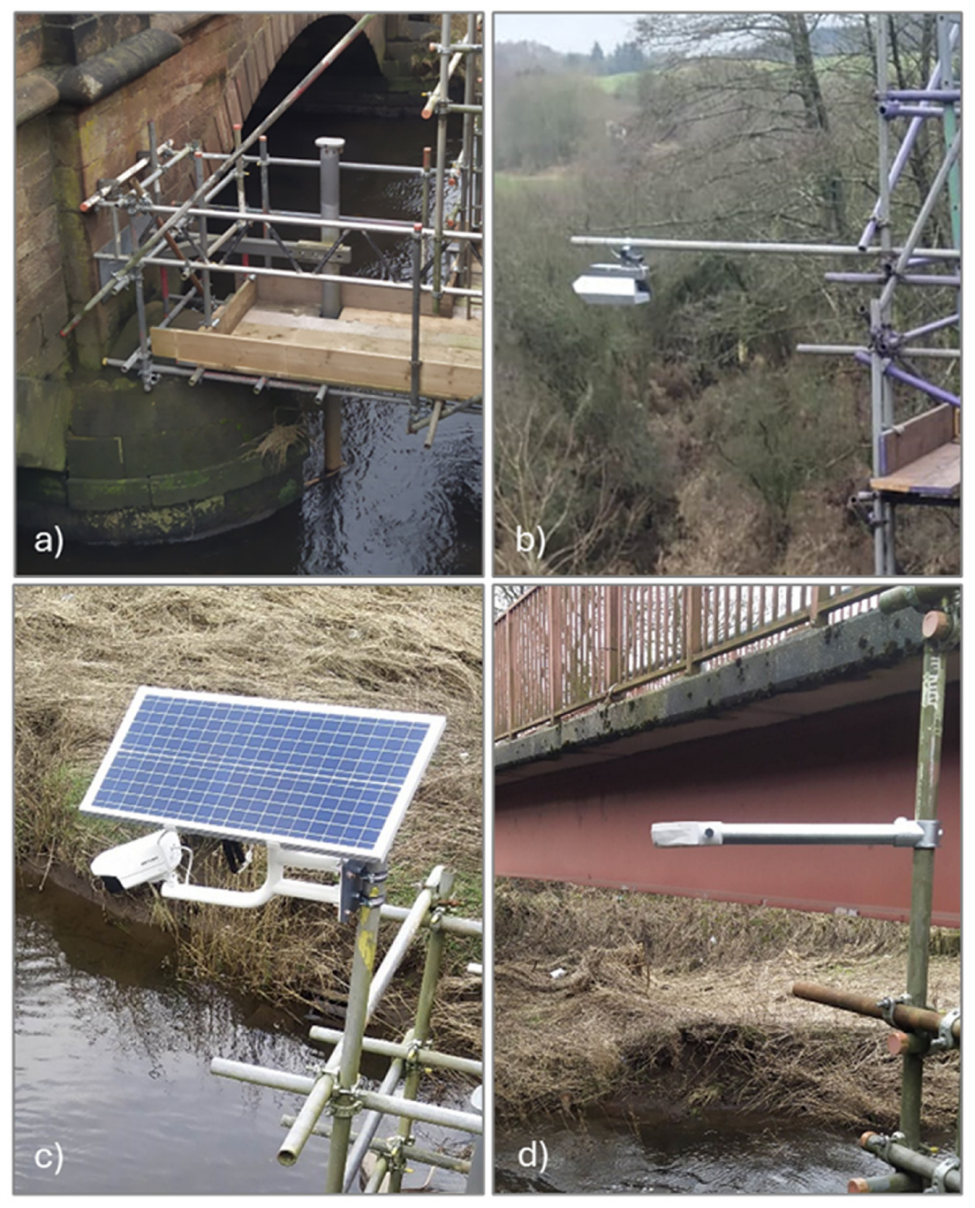
Collection of installed and tested sensors. **a**) EnviroSCAN Scour Probe. **b**) OTT radars. **c**) Solar camera. **d**) RiverTrack sensor.

***Scour Probes.*** Scour probes ( Figure 2. *a*) are devices equipped with electromagnetic sensors, commonly used for determining soil moisture. Prototypes of these probes have been employed in previous studies to monitor changes in scour near bridge piers ( Maroni *et al.*, 2020). Each sensor measures the resonant frequency of the surrounding medium, which is then converted into permittivity values, allowing the identification of the material around the sensor. Once calibrated, the probes can differentiate between air, water, soil, and potentially detect sediment deposition. By knowing the elevation of the sensors, both the water level and riverbed elevation can be determined. The measurements are limited by the number and positioning of the sensors, offering a resolution of approximately 10 cm. The data are localised, meaning they are measured at the location where the sensor is installed. For the results to be meaningful, the probes must be placed at locations corresponding to the maximum depth of the scour holes. Despite their ability to directly monitor the scour process, these probes are costly and challenging to install. Installation requires embedding the probes into the riverbed, which presents both technical challenges and permitting issues for access. The data collected from the Scour *p*robes will be used in subsequent studies to evaluate the indirect monitoring approach. 

***OTT Radar.*** Radars ( Figure 2.b) are commonly used in riverine hydraulic monitoring, although they are expensive devices. In the project, two sensors were considered: the Radar Level Sensor (RLS) for water depth and the Surface Velocity Radar (SVR) for velocity, both from OTT ( https://www.ott.com/products/water-level-1/ott-rls-radar-level-sensor-861/). This type of radar provides only an average velocity value over the sensor's footprint. While highly accurate, the measurements are localised. The installation, data transfer, and storage are managed by the supplier, and the system requires minimal maintenance.

***Solar Camera.*** Solar cameras are affordable, low-power sensor systems, typically used for surveillance, that can also be employed for monitoring river flows. The solar camera system consists of a photovoltaic panel, a battery, and an optical camera ( https://www.hikvision.com/en/products/IP-Products/Network-Cameras/solar-powered-security-camera-setup/ds-2xs6a25g0-i-ch20s40-no-battery/). For this project, the Hikvision DS-2XS6A25G0 camera was selected ( Figure 2.c). This optical sensor features a 2.8 mm focal length, records video at 2 MP resolution and 30 fps, and is equipped with infrared (IR) light for video capture in low-light conditions. The system utilises a 5G wireless network for data transmission and is capable of automatically recording short video clips. The use of these cameras requires post-processing of the recorded video. The camera is primarily used for image velocimetry, providing surface velocity and discharge measurements when the cross-section is available. While image velocimetry is widely used, long-term applications remain limited.

***River track.*** The RiverTrack sensors ( Figure 2.d) are affordable, low-power devices designed for local community flood alert systems ( https://www.rivertrack.org/). They can be connected to either the LoRaWAN or 5G network, providing real-time data. The water level measurement model, which features a 40kHz ultrasonic transducer, is used by several communities in the UK. The sensor provides precise measurements, is very cost-effective, and is easy to install. A version of the sensor, enhanced with an optical sensor to detect velocity, is currently under validation.

## 4 Pilot case studies

Two pilot case studies were established at the critical bridges identified in Section 3.1, where the selected sensors (described in Section 3.2) were installed to implement an indirect monitoring strategy. These case studies have already successfully collected valuable data and continue to operate effectively.

**Figure 3.**
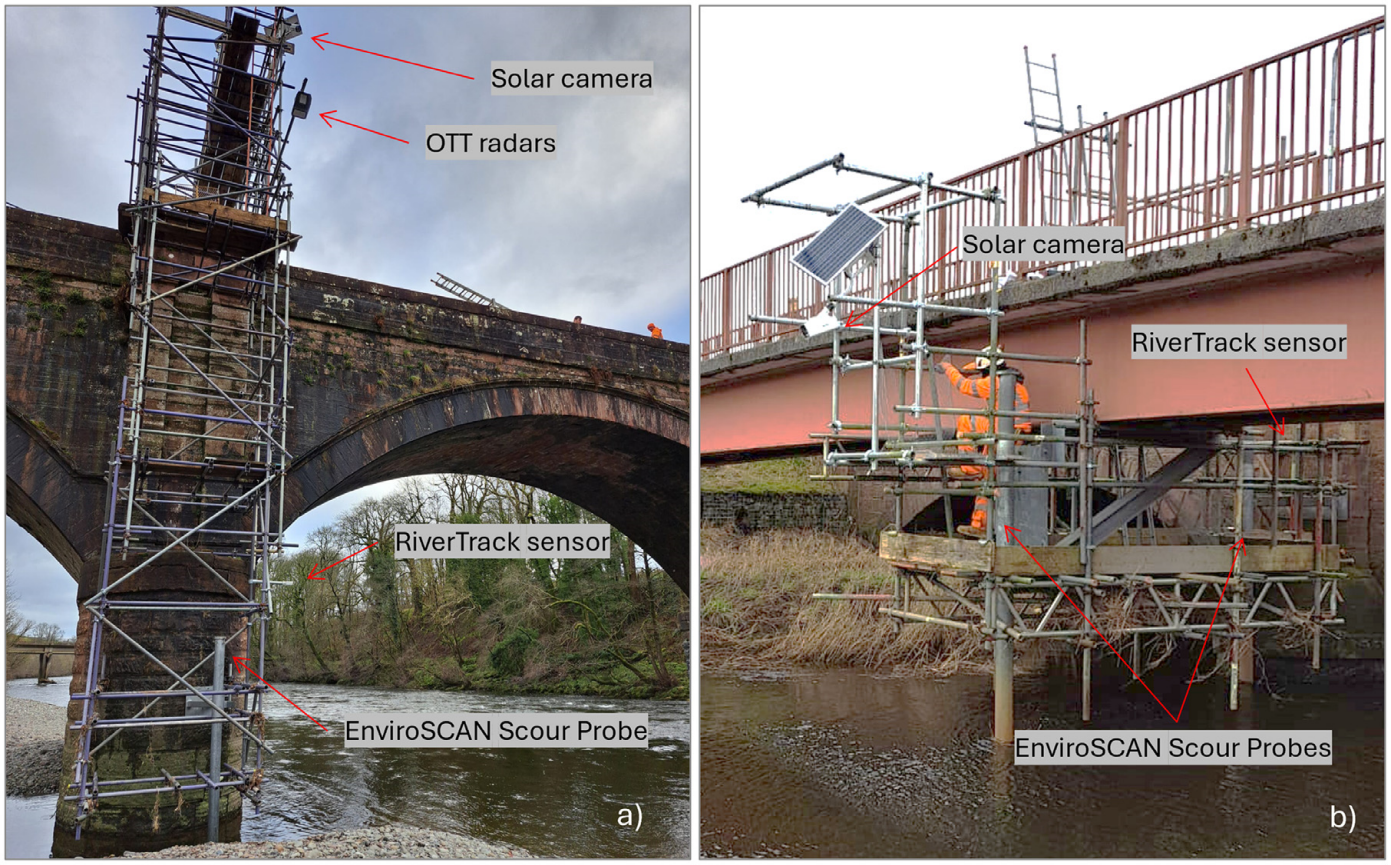
Overview of the two pilot cases with installed sensors. **a**) Auldgirth Old Bridge. **b**) New Cumnock Bridge.

At Auldgirth ( Figure 3.a), the focus is on the left pier due to its higher exposure to scour, while the presence of a rocky formation at the base of the right pier reduces its susceptibility. A scaffolding structure was erected to support the installation of the sensors in front of the left pier. The monitoring system deployed at this site includes a solar camera, a RiverTrack sensor, an OTT radar system with a velocity sensor and a water level sensor, as well as a scour probe. The camera captures the stretch of river upstream of the bridge, and a topographical survey of Ground Control Points was conducted on the day of installation to calibrate the camera and perform image orthorectification. Soil samples from the vicinity of the riverbed were also collected for sieve analysis to establish the grain size distribution curve for use in future analyses. The OTT radar, which measures water level, and the RiverTrack sensor are positioned perpendicular to the river surface near the left pier, while the velocity sensor of the OTT radar is directed at the area in front of the pier. However, the left pier is only partially within the riverbed, becoming fully submerged only during medium to high flow conditions. Moreover, the sensor is installed immediately downstream of a bend, which causes the footprint of the OTT velocity sensor to cover areas that are not consistently submerged. Consequently, velocity measurements are available only when the area is actually wet. The scour probe was placed at the centre of the scour hole to detect the maximum scour depth. Unfortunately, shortly after installation, during a significant flood, water entered the device, damaging it. This highlighted a practical issue with the proper sealing of the top cup. The sensor will be replaced with a new one, and to address the issue, the probe’s end will be raised to prevent submersion. Additionally, a new end cover design will be implemented to facilitate easier access to the probe and ensure proper sealing.

At New Cumnock ( Figure 3.b), in addition to the two previously installed scour probes, a RiverTrack sensor and a solar camera were mounted on the existing scaffolding. The RiverTrack sensor is positioned perpendicular to the river surface near the left pier, where one of the scour probes is also located. The camera is placed at the centre of the river's cross-section, facing the stretch of river immediately upstream of the bridge. At this site, the camera was frequently vandalised, and additional protective measures were implemented to safeguard it. Furthermore, on the day of the camera installation, a survey of Ground Control Points was carried out to calibrate the camera. Additionally, a soil sample was collected near the riverbed for sieve analysis to determine the grain size distribution curve, which will be used in subsequent analyses.

## 5 Evaluation of hydraulic monitoring strategies

The data recorded by the sensors at the two pilot case studies constitute an open-access dataset that provides valuable field data for further research. This dataset will be continuously updated, as the case studies are expected to remain active and contribute to a long-term data repository. A preliminary analysis of the hydraulic data collected so far has already revealed several important insights.

**Figure 4.**
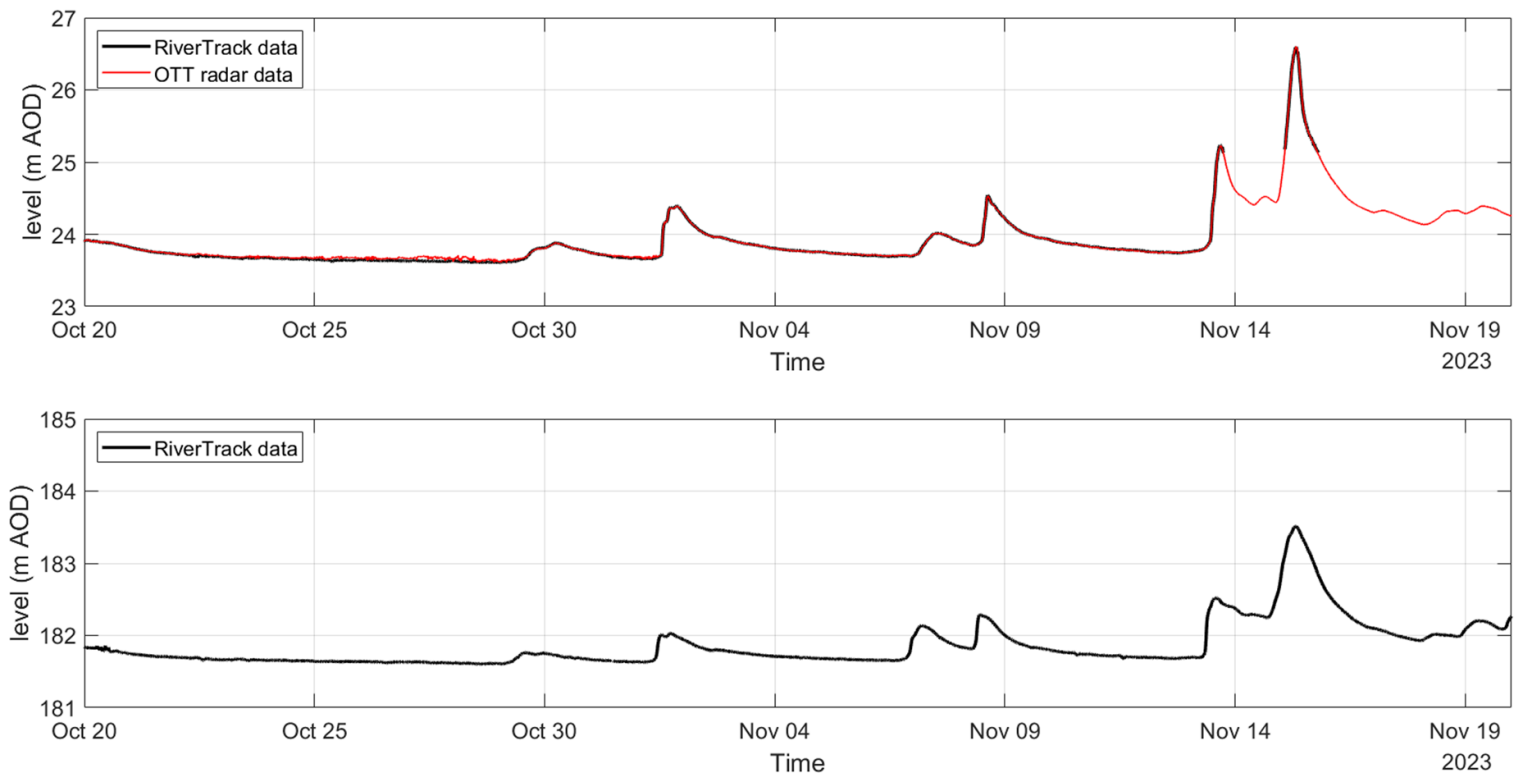
Water level monitoring using RiverTrack and OTT radar at Auldgirth Old Bridge (top panel) and New Cumnock Bridge (bottom panel).

The comparison between the OTT radar sensor (a high-cost solution) and the RiverTrack sensors (a low-cost alternative) highlighted the potential of economic monitoring tools. As shown in Figure 4, the RiverTrack sensor measured water level variations, expressed in mAOD (metres Above Ordnance Datum), with the same accuracy as the OTT radar sensor, while offering significant advantages in terms of cost. Additionally, the RiverTrack sensor is powered by simple lithium batteries, in contrast to the photovoltaic panels required by the OTT radar, and its compact design (12 × 12.3 × 5.1 cm) makes installation considerably easier. The absence of data observed in November for the RiverTrack sensor installed at Auldgirth ( Figure 4) is likely due to foliage or other flood debris becoming tangled in the scaffolding or bridge structure. This suggests that the sensor should be positioned at a sufficient distance from the structure to avoid interference with water level readings. Despite this minor issue, the results highlight the reliability and practicality of low-cost sensors like the RiverTrack for water level monitoring applications.

The video data captured by the cameras were analysed to monitor velocity and discharge in the upstream sections of the bridges. This approach enables distributed measurements across the entire river cross-section, offering a significant advantage over localised velocity measurements obtained from the SVR radar. By capturing spatially extensive data, this method reduces the uncertainty associated with assumptions regarding the horizontal velocity profile. The camera system continuously recorded short oblique videos of the river surface at a specified sampling frequency. To extract quantitative information from the optical data, it was essential to resolve the camera geometry by defining the camera matrix, a process that enabled the conversion of image coordinates into real-world coordinates and eliminated perspective distortion ( Bruder & Brodie, 2020). The camera matrix consists of two main components. The intrinsic parameters, such as focal length, zoom, and pixel scale, depend on the camera's specific features and were calibrated in the office using the open-source software Camera Calibration Toolbox for Matlab ( Bouguet, 2022). The extrinsic parameters, which describe the camera's position and orientation, required a field survey to identify ground control points directly at the site. This calibration process ensured accurate data extraction for subsequent analyses.

For the estimation of surface velocity, several image velocimetry technologies were tested, including Particle Tracking Velocimetry (PTV), Particle Image Velocimetry (PIV), and Space-Time Image Velocimetry (STIV). A test video was recorded at New Cumnock, accompanied by a simultaneous manual measurement of river velocity at a specific cross-section immediately upstream of the analysed bridge using a mechanical flowmeter. The results from different image velocimetry methods were compared with the measured data, leading to the selection of the Particle Image Velocimetry (PIV) approach. The comparison was conducted under low-flow conditions to allow safe access to the river for manual measurements. This also provided an opportunity to evaluate the technique under scenarios that are typically more challenging for such methodologies, particularly due to the difficulty in identifying flow patterns. PIV is an Eulerian technique commonly used to determine velocity at fixed spatial positions. Initially developed for laboratory analyses, PIV has been adapted over the years for field applications, evolving into Large-Scale Particle Image Velocimetry (LSPIV). It computes the displacements of surface flow features travelling along the river by evaluating the cross-correlation of small sub-images ( Hauet *et al.*, 2008), therefore, it requires the presence of visible patterns or floating objects on the water surface.

The displacement vectors, expressed in pixels per frame, were estimated using the PIVLab_2.59 code. To convert the field velocities into meters per second, the Rectification of Image Velocity Results (RIVeR_2.6) toolbox was employed, a standalone MATLAB application designed for large-scale water surface characterisation ( Patalano *et al.*, 2017). This approach allowed for the identification of the horizontal profile of surface velocity at a specific cross-section. Any data gaps in the horizontal profile were subsequently filled by the software, which applied an appropriate horizontal distribution to complete the velocity profile. The surface velocities ( *V _s_*) were then converted into mean stream velocity ( *V _m_*) using the alpha method, which defines the relationship between surface and mean velocity through a dimensionless coefficient (∝):


$$V_{m}=\propto V_{s}\;(1)$$

From the velocity survey conducted at New Cumnock, an alpha value of 0.9 was estimated. Discharge ( *Q*) was then calculated by multiplying the mean velocity by the wet area ( *A*).


$$Q=V_{m}A\;(2)$$

The water level data recorded by the RiverTrack sensor was used to calculate the wet area and wet perimeter.

**Figure 5.**
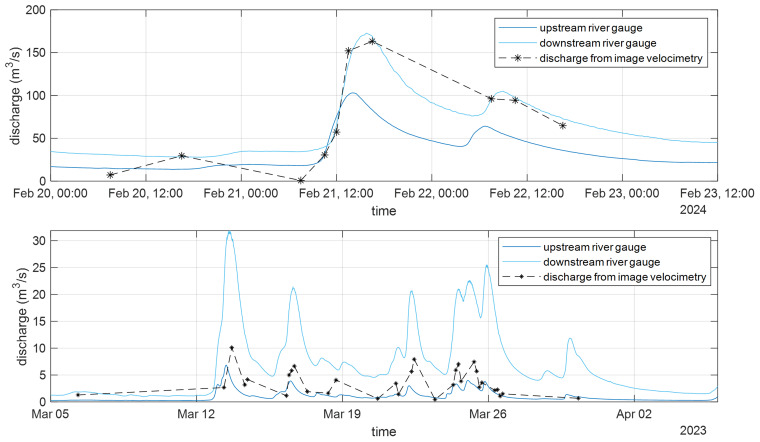
Discharge estimated from the cameras at Auldgirth Old Bridge (top panel) and New Cumnock Bridge (bottom panel, adapted from the paper included in the proceedings of the River Flow conference ( Perugini *et al.*, 2024)).

This analysis was repeated for all images where surface patterns were visible ( Figure 5). Not all available video footage could be used, as the methodology is sensitive to visibility conditions. Factors such as rain and wind can also affect the accuracy of the analysis. The discharges estimated from the suitable videos are shown in Figure 5 as asterisks. These data were then linearly interpolated to obtain the continuous time series of discharge estimated from the videos. This video-derived discharge time series was validated against the discharge data provided by SEPA for the nearby upstream and downstream gauges (refer to Figure 1 for the location of the river gauges). The results showed that the temporal variation in discharge and the identification of flood events were well estimated. A correlation analysis was also performed to evaluate the reliability of the estimated data (see also Perugini *et al*., 2024), confirming that image velocimetry results can be used to characterise the river properties at the Nith Bridge, even if further validation and comparison with measured data at the bridge are required. Additionally, further analysis is necessary to better understand the influence of uncertainty in the alpha parameter.

To enable the adoption of long-term applications, it is essential to advance the automation of the analysis process, minimising manual inputs during post-processing and improving the handling of unreliable results. This refinement is particularly important as the methodology is highly sensitive to environmental conditions such as lighting, rain, and wind. Nonetheless, the results obtained so far are highly encouraging and warrant deeper investigation into this research area.

## 6 Conclusion

The findings of this study underscore the critical importance of effective bridge scour monitoring strategies in enhancing the resilience of transportation infrastructure against hydraulic hazards and highlight the need for innovative, cost-effective monitoring solutions. The comparative analysis between the OTT radar system and the RiverTrack sensors demonstrates that low-cost monitoring solutions can achieve comparable accuracy in measuring water levels, offering a viable alternative to more expensive technologies. These solutions also have the advantage of being easier to install and having low energy consumption, making them sustainable tools. Furthermore, they can transmit data remotely, which is essential for enabling real-time monitoring.

Moreover, the use of cameras for image velocimetry should be further promoted, as they represent an effective low-cost tool capable of capturing spatially extensive velocity measurements across the river's cross-section, significantly reducing the uncertainties associated with localised measurements. However, the installation of cameras is often vulnerable to vandalism; therefore, involving the local community in preventing such incidents and installing protective measures is crucial. Additionally, further research is needed to make this technology more user-friendly for long-term use.

Lastly, it is important to emphasise that for a successful monitoring system, proper data management must be defined in advance, as sensors will collect large volumes of data that need to be georeferenced and synchronised.

In conclusion, this work contributes to addressing the pressing need for robust monitoring strategies to assess bridge scour risks. The findings emphasise the importance of effective monitoring in capturing the complexity of real-world conditions..

## Ethics and consent

Ethics and consent were not required.

## Data Availability

The dataset recorded from the two case studies is progressively updated due to the continuous nature of the monitoring strategy and the most recent version can be found in the following repository. zenodo: “Two pilot case studies for bridge-scour monitoring” RAMOBRIS_field_dataset https://doi.org/10.5281/zenodo.14237317. ( Perugini, 2024) The dataset contains the following underlying data: • README.pdf. (This document describes the structure of the dataset and all the files included.) • Hydraulic monitoring (Folder containing the dataset, including the raw and elaborated data for both pilot case studies.) Data are available under the terms of the Creative Commons Attribution 4.0 International license (CC-BY 4.0)
