# Supplementary material for: Two pilot case studies for indirect bridge scour monitoring using low-cost remote sensing for river flow characterisations
Source: Open Res Eur. 2025 Nov 28;4:274. Originally published 2024 Dec 24. [Version 2] doi: 10.12688/openreseurope.19083.2 (PMC12744198; doi:10.12688/openreseurope.19083.2)
Supplement: Peer review history

# Reviewer report: Reviewer response for version 2

**DOI:** 10.21956/openreseurope.23578.r65443

**Published:** 2026-01-05

**Authors:** Francesco Ballio

**Affiliations:** 1 Politecnico di Milano, Milano, Italy

The manuscript discuss some strategies for bridge hydraulic monitoring, with particular focus on water depth and velocity. The final aim of the project would be to use flow variablesn  as control parameters of scour models, thus inferring local scour (at piers) without direct measurement.

 Conclusions from present studies are only partial, in that the feasibility of deriving reliable estimates of scour depths from flow variables is not proven. However, due to the strong interest of the bridge managers in hydraulic monitoring approaches, results from the two field cases discussed in the manuscript are surely interesting and valuable.

 As a general comment I would recommend authors to better clarify their strategy of “indirect bridge scour monitoring”: in facts, no such result is here shown, nor it is made explicit how scour values should be derived from measured quantities; on the other hand, field installations comprehend scour sensors (for direct monitoring), but the role of such sensors is not specified, nor measured data are shown.

 Some more specific comments follow (all related to the “introduction” section).

 1. Contraction scour is not only due to the “presence of bridge piers or abutments”. It can also be due to (i) embankments and (ii) natural narrowing of the river channel or overbank areas.

 2. Local scour is not only due to ”turbulence and vorticity that increase shear stress”. The very first effect of the presence of a pier (or abutment in the flow) is to locally increase the time-averaged velocity and, consequently, the shear stress.

 3. When describing the process of scour holes more or less gradually deepening in time, authors should also mention that such holes tend to be refilled in the aftermath of hydraulics events.

Is the case presented with sufficient detail to be useful for teaching or other practitioners?

Yes

Is the work clearly and accurately presented and does it cite the current literature?

Yes

If applicable, is the statistical analysis and its interpretation appropriate?

Not applicable

Are all the source data underlying the results available to ensure full reproducibility?

Yes

Are the conclusions drawn adequately supported by the results?

Yes

Is the background of the case’s history and progression described in sufficient detail?

Yes

Reviewer Expertise:

River hydraulics, scour at bridges

I confirm that I have read this submission and believe that I have an appropriate level of expertise to confirm that it is of an acceptable scientific standard, however I have significant reservations, as outlined above.

# Reviewer report: Reviewer response for version 2

**DOI:** 10.21956/openreseurope.23578.r65018

**Published:** 2025-12-27

**Authors:** José C Matos, Sinem Tola

**Affiliations:** 1 University of Minho, Guimarães, Portugal; 2 ISISE-Civil Engineering, Universidade do Minho Departamento de Engenharia Civil (Ringgold ID: 549494), Guimaraes, Braga, Portugal

Dear Dr Perugini and co-authors,

 We have checked the changes against my previous minor comments. I am pleased to confirm that all points have been properly addressed: the revised title and expanded context in the early sections improve the framing and relevance, the figure references and caption formatting have been corrected, the monitoring setup is now clearly illustrated, and the methodological section is more complete with the added formulations and clearer discussion of the results.

 Thank you.

 Kind regards,

 Sinem Tola & Jose C. Matos

Is the case presented with sufficient detail to be useful for teaching or other practitioners?

Yes

Is the work clearly and accurately presented and does it cite the current literature?

Partly

If applicable, is the statistical analysis and its interpretation appropriate?

Not applicable

Are all the source data underlying the results available to ensure full reproducibility?

Yes

Are the conclusions drawn adequately supported by the results?

Yes

Is the background of the case’s history and progression described in sufficient detail?

Yes

Reviewer Expertise:

Bridge scour detection using drive-by monitoring signals

We confirm that we have read this submission and believe that we have an appropriate level of expertise to confirm that it is of an acceptable scientific standard.

# Reviewer report: Reviewer response for version 2

**DOI:** 10.21956/openreseurope.23578.r65017

**Published:** 2025-12-11

**Authors:** Manu Sasidharan

**Affiliations:** 1 Civil, Environmental and Geomatics Engineering, University College London (Ringgold ID: 4919), London, UK

The authors have revised and updated the manuscript and can be indexed.

Is the case presented with sufficient detail to be useful for teaching or other practitioners?

Yes

Is the work clearly and accurately presented and does it cite the current literature?

Partly

If applicable, is the statistical analysis and its interpretation appropriate?

Yes

Are all the source data underlying the results available to ensure full reproducibility?

Yes

Are the conclusions drawn adequately supported by the results?

Yes

Is the background of the case’s history and progression described in sufficient detail?

Yes

Reviewer Expertise:

Infrastructure asset management, bridge scour management, risk modelling, climate resilience

I confirm that I have read this submission and believe that I have an appropriate level of expertise to confirm that it is of an acceptable scientific standard.

# Reviewer report: Reviewer response for version 1

**DOI:** 10.21956/openreseurope.20649.r49272

**Published:** 2025-01-25

**Authors:** José C Matos, Sinem Tola

**Affiliations:** 1 University of Minho, Guimarães, Portugal; 2 ISISE-Civil Engineering, Universidade do Minho Departamento de Engenharia Civil (Ringgold ID: 549494), Guimaraes, Braga, Portugal

Manuscript: Two pilot case studies for bridge-scour monitoring.

Summary: The research proposes an affordable scour monitoring based on the acquisition of flow characteristics using video data acquired by solar cameras and the use of acquired hydraulic data to estimate scour depth with formulas. The superiority of the proposed monitoring equipment is its lower cost and ease of installation compared to OTT radars. The researchers achieved accuracy comparable to OTT radars.

Recommendation:  Accept with minor revisions

1. The title is concise but should include further information regarding the specific scour monitoring method employed, i.e., image velocimetry techniques.
2. The introduction provides a comprehensive overview; however, it would be beneficial to incorporate a concise discussion of the study's practical implications at the outset.
3. In Section 2, the second secondary argument of the first paragraph, i.e., the expression "Secondly, the availability of high-quality monitoring data is fundamental for calibrating and validating numerical models, as well as for training and testing AI-based methods," can be enriched by the addition of references to previous research.
4. The last paragraph of Section 2 discusses the bridge monitoring approaches. A couple of references to former research could be added for the mentioned methods. Specifically, the presentation of the limitations of a former radar-based scour monitoring research study could provide a very supportive argument for the proposed methodology using lower-cost monitoring.
5. It appears that some of the details of the case studies provided in Section 3.1 are more suitably placed in Section 4. Therefore, a minor reorganization of the information may enhance the research narrative. It is recommended that Section 3 be initiated with an introductory paragraph that provides a concise overview of the methodological steps (including data processing) before the subsections.
6. In Section 3.2, the reference figure for River Track sensors is Figure 2.d instead of Figure 1.d; the reference figure for OTT Radars is Figure 2.b instead of Figure 1.b; the reference figure for the solar camera is Figure 2.c instead of Figure 1.c.
7. Section 3.2 references the first case study in Figure 1.b instead of Figure 2.b.
8. In Section 3.2, additional clarification regarding the concept of "localized data" could facilitate a more lucid reading of the text.
9. The captions of Figures are not consistently rendered in bold; this practice should be uniform.
10. The slight blurring observed in Figures 5 and 6 may be attributed to the printing of the document as a Portable Document Format (PDF); however, higher resolutions are recommended for optimal visual clarity.
11. In Section 4, at the following expression: “Additionally, due to the bridge’s position immediately downstream of a bend, the radar often pointed to regions that were not consistently submerged, leading to occasional inaccuracies in velocity measurements. “ The presence of such inaccuracies is acknowledged, and it is recommended that their extent be specified.
12. It is recommended that a sketch be included in Section 4 to illustrate the positioning of the monitoring equipment.
13. The unit for the level of Figure 4 should be clarified since the notation mAOD isn’t explicitly defined in the text.
14. The numerical results that Figure 5 reveals should be discussed more in the text.
15. Section 5 could be made more explicit in presenting formulations such as the alpha method.

Is the case presented with sufficient detail to be useful for teaching or other practitioners?

Yes

Is the work clearly and accurately presented and does it cite the current literature?

Partly

If applicable, is the statistical analysis and its interpretation appropriate?

Not applicable

Are all the source data underlying the results available to ensure full reproducibility?

Yes

Are the conclusions drawn adequately supported by the results?

Yes

Is the background of the case’s history and progression described in sufficient detail?

Yes

Reviewer Expertise:

Bridge scour detection using drive-by monitoring signals

We confirm that we have read this submission and believe that we have an appropriate level of expertise to confirm that it is of an acceptable scientific standard, however we have significant reservations, as outlined above.

# Response

**Published:** 2025-10-30

**Authors:** Eleonora Perugini

**Affiliations:** Civil & Environmental Engineering, University of Strathclyde, Glasgow, Scotland, UK

We thank the reviewers for the constructive feedback and the opportunity to improve our manuscript. We have carefully addressed all points and revised the manuscript accordingly. Below, we provide a detailed point-by-point response. We appreciate the opportunity to enhance the clarity and robustness of our study and hope the revised version meets your expectations.  

1. We have revised the title to include additional information on the specific scour monitoring method employed. The new title is: “Two pilot case studies for indirect bridge scour monitoring using low-cost remote sensing for river flow characterisation.”
2. A brief discussion of the study's practical implications has been added to Section 1, stating: “The outcomes of the study support the implementation of scour monitoring systems, contributing to improved flood risk management, more informed decision-making, and enhanced early warning capabilities, while strengthening the resilience and security of critical infrastructure.”
3. The reference to previous research has been added as requested.
4. In the revised version, we have included references to previous research that offer an overview of the current state of the art and limitations in scour monitoring techniques.
5. Section 3.1 presents the two selected case studies, outlining their main characteristics and the rationale for their selection. Section 4, instead, provides a detailed description of the monitoring systems implemented at the two sites, which were adapted to the site-specific features of each bridge. This distinction between the two sections has been made clearer in the revised introduction of Section 3. Moreover, the introduction of Section 3 has been rewritten to provide a concise overview of the methodological steps. The revised text now reads as follows: “The methodological approach adopted in this study began with the identification of suitable bridge sites and the selection of appropriate monitoring technologies. After a thorough search for potential locations and a review of available sensors, two bridges were selected as pilot sites based on their structural characteristics and susceptibility to scour (Section 3.1). The most suitable instruments were then chosen according to the specific monitoring objectives and site constraints. Following this preliminary assessment, the sensors described in Section 3.2 were installed to establish two pilot case studies (Section 4), enabling continuous monitoring of the hydraulic and morphological conditions at the selected bridges. The implemented systems were designed to measure parameters such as water level, flow velocity, and bed elevation, providing real-time information on scour processes under varying hydraulic conditions, and to test both low-cost and more expensive sensor technologies. Subsequently, the data collected from the monitoring systems were processed and compared in order to evaluate the performance of the installed technologies.”
6. Thank you for this observation. The references to the figures have been checked and corrected.
7. The reference has been checked and corrected.
8. By “localized data” we mean measurements obtained at a specific location, as in the case of scour probes, or averaged over the footprint of the sensor, as in the case of RiverTrack or OTT radar. This differs from data acquired by solar cameras, which cover the entire cross-section or a portion of the river. This distinction has been clarified in the text.
9. The formatting of the figure captions has been updated.
10. We attempted to enhance the quality of the images.
11. We have rewritten the sentence to clarify the issue. The new sentence is: “However, the left pier is only partially within the riverbed, becoming fully submerged only during medium to high flow conditions. Moreover, the sensor is installed immediately downstream of a bend, which causes the footprint of the OTT velocity sensor to cover areas that are not consistently submerged. Consequently, velocity measurements are available only when the area is actually wet.”
12. The positioning of the monitoring equipment has been illustrated and specified in Figure 3.
13. The unit mAOD (metres Above Ordnance Datum) has now been explicitly defined in the text.
14. The results derived from image velocimetry and shown in Figure 5 have been better described, and the following sentence has been added to the text: “The discharges estimated from the suitable videos are shown in Figure 5 as asterisks. These data were then linearly interpolated to obtain the continuous time series of discharge estimated from the videos.”
15. The formulas have been specified in the text.

# Reviewer report: Reviewer response for version 1

**DOI:** 10.21956/openreseurope.20649.r49274

**Published:** 2025-01-21

**Authors:** Manu Sasidharan

**Affiliations:** 1 Civil, Environmental and Geomatics Engineering, University College London (Ringgold ID: 4919), London, UK

The paper reports on two pilot case studies for bridge scour monitoring and underscores the necessity of such measures for managing bridge scour. The discussions and findings from the paper is academically sound and serves as an enabler for developing business cases for scour monitoring.

 Considering that the paper adopts the approach of measuring the river flow properties, the literature review of time-dependent scour prediction models that employ such datasets to predict the occurrence or progression of scouring is missing. Recent research such as [Ref 1], [Ref 2] and [Ref 3] etc. have proposed different models that employs hydraulic data for modelling scour progression. The literature review within this manuscript needs to capture this effectively.

Is the case presented with sufficient detail to be useful for teaching or other practitioners?

Yes

Is the work clearly and accurately presented and does it cite the current literature?

Partly

If applicable, is the statistical analysis and its interpretation appropriate?

Yes

Are all the source data underlying the results available to ensure full reproducibility?

Yes

Are the conclusions drawn adequately supported by the results?

Yes

Is the background of the case’s history and progression described in sufficient detail?

Yes

Reviewer Expertise:

Infrastructure asset management, bridge scour management, risk modelling, climate resilience

I confirm that I have read this submission and believe that I have an appropriate level of expertise to confirm that it is of an acceptable scientific standard.

## References

[1] : Estimation of time dependent scour depth around circular bridge piers: Application of ensemble machine learning methods. *Ocean Engineering* .2023;270: 10.1016/j.oceaneng.2022.113611 10.1016/j.oceaneng.2022.113611

[2] : Comparison of hybrid deep learning models for estimation of the time-dependent scour depth downstream of river training structures. *Physics of Fluids* .2024;36(10) : 10.1063/5.0231597 10.1063/5.0231597

[3] : A bridge scour risk management approach to deal with uncertain climate future. *Transportation Research Part D: Transport and Environment* .2023;114: 10.1016/j.trd.2022.103567 10.1016/j.trd.2022.103567

# Response

**Published:** 2025-10-30

**Authors:** Eleonora Perugini

**Affiliations:** Civil & Environmental Engineering, University of Strathclyde, Glasgow, Scotland, UK

We thank the reviewer for their valuable comments and suggestions, which have helped us to improve the manuscript. Accordingly, the literature review has been expanded in the second version of the manuscript, and the following paragraph has been added to Section 2: “Another method is to directly measure flow properties, such as water level and velocity, and use these data as input to estimate scour depth through advanced scour formulas or artificial intelligence techniques, thereby indirectly determining the scour depth. Perugini et al. (2024) presented a proof of concept for near real-time estimation of scour at a bridge pier by combining low-cost sensors, large-scale image velocimetry techniques, and advanced scour models. Specifically, the flow properties collected by the sensors were used as input for a temporal scour model based on the dimensionless effective flow work (Link et al., 2020; Pizarro et al., 2017; Pizarro et al., 2022). Kumar et al. (2023) evaluated the performance of two ensemble methods and one standalone machine learning method for predicting time-dependent scour using laboratory data. Similarly, Azman et al. (2024) employed experimental data and deep learning algorithms to predict the evolution of scour depth downstream of a submerged weir.”
